# Differentially expressed genes for aggressive pecking behaviour in laying hens

**DOI:** 10.1186/1471-2164-10-544

**Published:** 2009-11-19

**Authors:** Bart Buitenhuis, Jakob Hedegaard, Luc Janss, Peter Sørensen

**Affiliations:** 1 Aarhus University, Faculty of Agricultural Sciences, Department of Genetics and Biotechnology, Blichers allée 20, P.O.Box 50, DK-8830 Tjele, Denmark

## Abstract

**Background:**

Aggressive behaviour is an important aspect in the daily lives of animals living in groups. Aggressive animals have advantages, such as better access to food or territories, and they produce more offspring than low ranking animals. The social hierarchy in chickens is measured using the 'pecking order' concept, which counts the number of aggressive pecks given and received. To date, little is known about the underlying genetics of the 'pecking order'.

**Results:**

A total of 60 hens from a high feather pecking selection line were divided into three groups: only receivers (R), only peckers (P) and mixed peckers and receivers (P&R). In comparing the R and P groups, we observed that there were 40 differentially expressed genes [false discovery rate (FDR) *P *< 0.10]. It was not fully clear how the 40 genes regulated aggressive behaviour; however, gene set analysis detected a number of GO identifiers, which were potentially involved in aggressive behavioural processes. These genes code for synaptosomes (GO:0019797), and proteins involved in the regulation of the excitatory postsynaptic membrane potential (GO:0060079), the regulation of the membrane potential (GO:0042391), and glutamate receptor binding (GO:0035254).

**Conclusion:**

In conclusion, our study provides new insights into which genes are involved in aggressive behaviours in chickens. Pecking and receiving hens exhibited different gene expression profiles in their brains. Following confirmation, the identification of differentially expressed genes may elucidate how the pecking order forms in laying hens at a molecular level.

## Background

Aggressive behaviour in group-living animals is an important aspect of their daily lives, and this behaviour is partly used to establish social ranks in groups. Animals who rank highly in the social hierarchy have many advantages, such as better access to food and territories [1,2]. In studies in chickens, highly ranked males mated more often and produced more offspring than low ranking males [3]. Likewise, dominant hens produced more offspring over their lifespan than sub-ordinate hens [4].

The social hierarchy in chickens can be measured by the number of aggressive pecks, which are usually aimed at the head of a receiving bird [5]. The onset of aggressive pecking differs between male and female chickens. Males initiate aggressive pecking behaviour in their second week after hatching, and the pecking reaches adult levels when the chicken are eight to nine weeks old. Females initiate aggressive pecks at approximately five weeks of age, and they reach adult levels at nine to 10 weeks of age [6-8]. A stable hierarchy is established at approximately 20 weeks of age, and a number of different factors are involved in its formation. Kim and Zuk [9] demonstrated that previous social experience, parasite status, morphological characteristics and possibly age can be important factors in establishing a hen's rank in the group.

In the European Union, poultry are commonly housed in free range housing systems (Directive 1999/74/EC). Aggression can be a problem in these flocks and result in increased social stress. Additionally, skin damage can trigger cannibalism. The level of aggression has been shown to be lower in large groups of chickens than in small groups [10]. In order to reduce aggressive encounters under practical settings, it is important to identify the genes involved in aggressive pecking behaviour to understand how the pecking order is established in chickens.

To date, little is known about the underlying genetic mechanisms behind aggressive pecking in chickens. Previous selection experiments showed that aggressive pecking was not related to feather pecking because while the propensity to peck feathers changed during selection, there was no effect on the aggressive pecking behaviour (reviewed in [11]). There are indications that 'group selection' experiments for high and low production and survivability can influence aggressive behaviour in laying hens [12]. Later studies on these selection lines demonstrated that there were changes in the dopaminergic and serotonergic systems [13]. Animals injected with dopamine D2 receptor blockers showed a reduced frequency of aggressive pecks on subordinates [14]. Administration of 5-HT1-A and 5HT1-B antagonists resulted in increased aggressive pecks depending on the selection line [15]. Both the dopaminergic and serotonergic systems have been shown to influence aggressive behaviours in both mammals and birds [16-18].

The present study aimed to identify genes that regulate the aggressive pecking behaviour in chickens. In order to identify these genes, we compared the genome-wide profiles of chicken brain samples from aggressive and receiver hens using a 20 K chicken microarray. We tested the hypotheses that (1) differentially expressed (DE) genes are associated with the number of aggressive pecks given or received and (2) genes are DE among peckers, receivers and a mixed group of peckers and receivers.

## Results

### Phenotype

**Figure 1:**
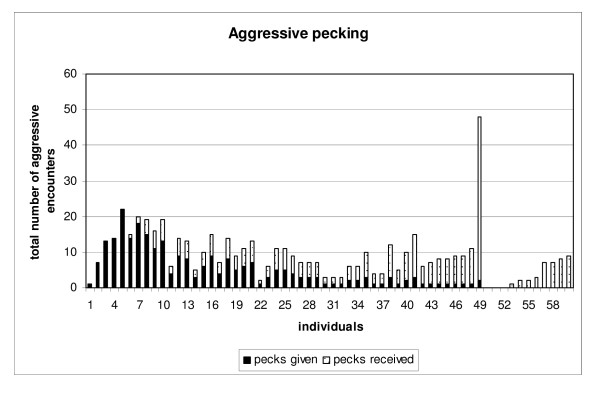
**Histogram showing the number of aggressive pecks given and received by each hen during a 3 h social test**.

The number of pecks given and pecks received per hen is shown in Figure 1. The number of pecks given during a three hour period ranged from 0 to 22, and the number of pecks received ranged from 0 to 46 (Figure 1). There was no difference between the cages in terms of the number of aggressive pecks performed per bird (Kruskal-Wallis χ^2^_2 _= 0.66, *P *= 0.72) or in the number of pecks received per bird (Kruskal-Wallis χ^2^_2 _= 1.34, *P *= 0.51). Additionally, there was no difference in the animal weights between the cages (F^1^_58 _= 0.64, *P *= 0.44).

### Number of pecks and gene expression

There was no relationship between the number of aggressive pecks given or received and the gene expression. The Spearman correlation ranged from -0.6 to 0.6, but the *P *value was between 0.99 and 1.

### Grouping of animals according to the pecks performed and received

Group 1 consisted of 44 animals (peckers and receivers, P&R), Group 2 consisted of eight animals (receivers, R), Group 3 consisted of five animals (peckers, P), and Group 4 consisted of three animals. Group 4 was considered too small to perform a gene expression experiment on, so it was not used. There was no difference in body weight between the groups (F^1^_58 _= 0.39, *P *= 0.54).

### Gene expression analysis

**Figure 2:**
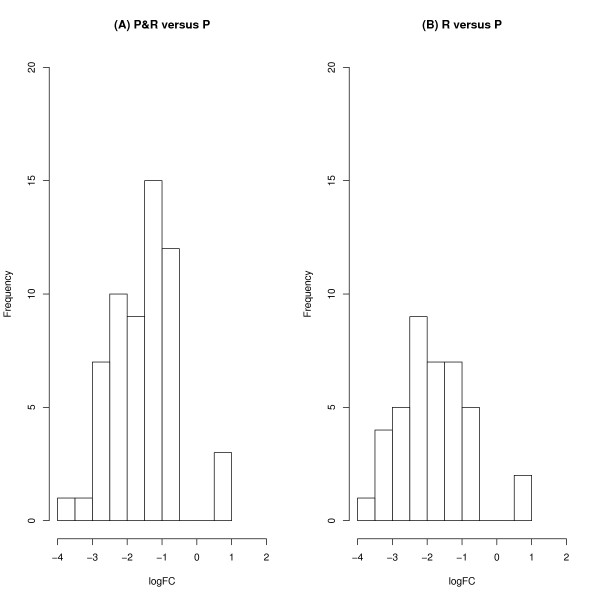
**Histogram of the log fold changes (logFC) of the significantly (FDR *P *< 0.1) differentially expressed genes in group comparisons**. Comparisons were made between (A) the pecker & receiver (P&R) and pecker (P) group, and between (B) the receiver (R) and the pecker (P) group.

**Figure 3:**
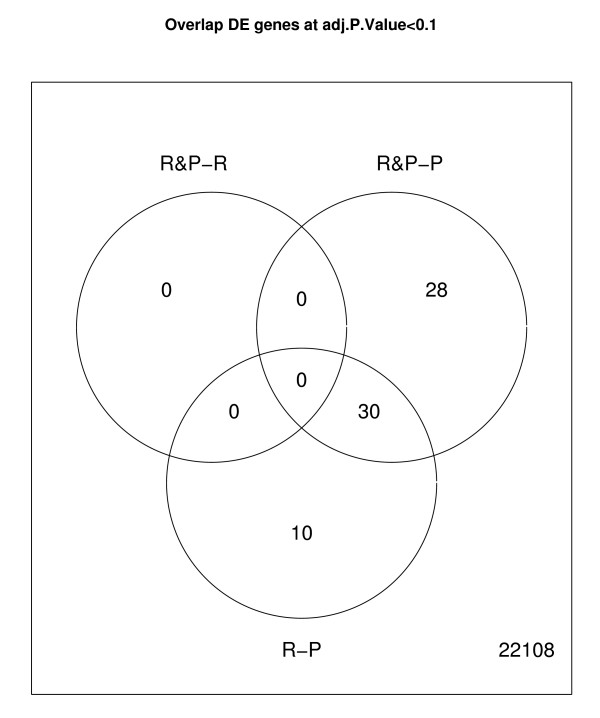
**Overlap of the significantly (FDR *P *< 0.1) differentially expressed genes between group comparisons**. Venn diagram of the pecker & receiver (P&R) vs receiver (R) group, the P&R vs pecker (P) group, and the R vs P group.

In Additional File 1, the DE genes (n = 179) for the comparison between P&R and R are presented. The logFC was in the range of 1.5 to -2.5. Of the 179 genes, 106 had a gene annotation, and 148 were mapped to the chicken genome. However, none of these genes were significant at the false discovery rate (FDR) *P *< 0.10. In Additional File 2, the genes (n = 342) for the comparison between P&R and P are presented. The logFC was in the range of 1 to -4. Of the 342 genes, 218 had a gene annotation, 300 were mapped to the chicken genome, and 58 were significant at the FDR *P *< 0.10. In Additional File 3, the genes (n = 337) of interest from the comparison between R and P are presented. The logFC was in the range of 1.5 to -4. Of the 337 genes, 208 had a gene annotation, 301 were mapped to the chicken genome, and 40 were significant at the FDR *P *< 0.10. Figure 2 shows the logFC distribution of the significant genes for the P&R vs. P and R vs. P comparisons. Figure 3 shows a Venn diagram of the overlapping genes in the comparisons. There were 30 genes in common between the P&R vs. P and R vs. P comparisons.

**Figure 4:**
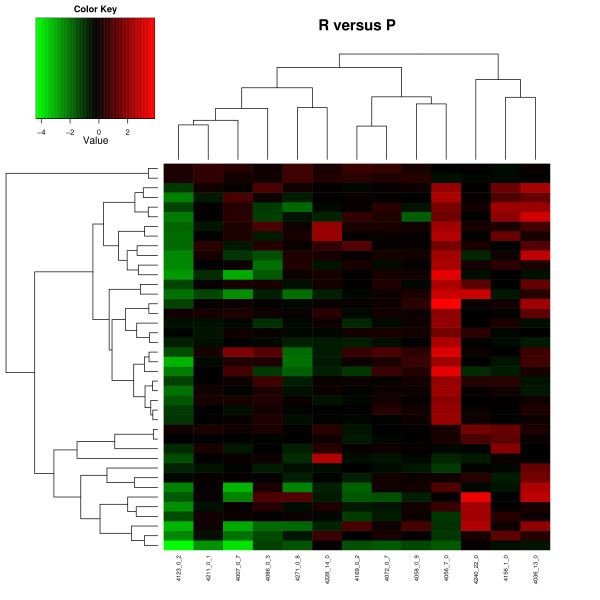
**Heatmap of differentially expressed genes between the receiver (R) and pecker (P) group**. There were 40 differentially expressed genes (FDR *P *< 0.1). The M values of the genes (rows) were ordered using the centred Pearson correlation and hierarchical clustering. The dendrogram shows the clustering results of the gene expression profiles. The arrays (columns) represent the individual hens, which are denoted with their id number, the number of aggressive pecks performed, and the number of aggressive pecks received (id_# pecks performed_# pecks received). The dendrogram shows the clustering results of hens based on the gene expression profiles. The red and green colours denote high and low intensities, respectively.

A heatmap of the 40 DE genes in the R vs. P comparison is shown in Figure 4. Clustering of the individuals based on their DE genes (FDR *P *< 0.10) showed that three out of the five peckers clustered together. The other two animals were assigned to two different clusters that contained receiver animals. Clustering of the individuals in the P&R vs. P comparison showed that the animals were mixed (see Additional File 4: Figure S1).

**Figure 5:**
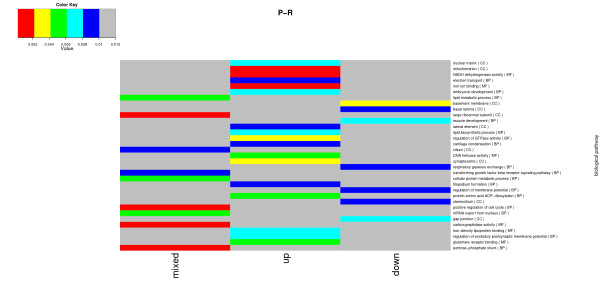
**Representation of the significant GO identifiers detected in the comparison between the receiver (R) and pecker (P) group**. The three alternatives (mixed, up, and down) are represented in the columns. The "mixed" alternative tested whether the genes in the set tended to be differentially expressed, without regard for the direction. In this case, the test is significant if the set mainly contains large test statistics, even if some results are positive and some are negative. The "up" alternative tested whether the genes in the set were up-regulated. The "down" alternative tested whether the genes in the set were down-regulated. The description of the biological pathways are listed in the rows and the GO class is listed in brackets (BP: biological processes, CC: cellular components, MF: molecular function). The colour gray denotes the GO identifiers *P *> 0.01.

We tested for overrepresentation of gene sets representing biological processes (BP), cellular components (CC), and the molecular function (MF) of gene ontology (GO) in the P&R vs. P and R vs. P comparisons. For the R vs. P comparison, 33 GO identifiers were significant (P < 0.01). Of these 33 GO identifiers, 17 belonged to the BP set, 10 belonged to the CC set, and six belonged to the MF gene set (Figure 5). The GO identifiers that could potentially be involved in behavioural processes were related to synaptosomes (GO:0019797), the regulation of excitatory postsynaptic membrane potential (GO:0060079), the regulation of membrane potential (GO:0042391), and glutamate receptor binding (GO:0035254). The GO:0019797, GO:0060079, GO:0035254 genes were the glutamate receptors (*GRIN1*, *GRIN2A *and *GRIN2B*). The GO:0042391 genes were mainly acetylcholine receptors (*CHRNA1*, *CHRNA3*, *CHRNA4*, *CHRNB4*). GO identifiers that were involved in muscle development and lipid biosynthesis were also identified. The GO identifiers detected from the P&R vs. P comparison are shown in Additional File 5: Figure S2.

## Discussion

This study identified genes that are involved in aggressive pecking behaviour in chickens, which is a behaviour related to social dominance. This research will help gain a better understanding of the underlying genetics of this behaviour. Our results showed that there was no association between number of pecks given or received and the gene expression level. However, comparison between the pecker and receiver animals showed that some genes were differentially expressed in the two groups.

### Grouping of animals

The aim of this study was to detect differences between pecker and receiver animals, and we assumed that the peckers were the aggressive animals and the receivers were the submissive animals or victims. In our study, we clearly divided groups of pecker (P) and receiver (R) animals. We are aware that there are animals in the pecker and receiver (P&R) group that have phenotypic profiles that may match either the pecker group or receiver group (Figure 1). However, it is difficult to assign a clear cut-off point to define an animal as a pecker or receiver when it both performs and receives aggressive pecks. Some researchers have used aggressive encounters (both pecks and receiving pecks) to rank animals in a social dominance hierarchy [19-22]. These approaches have varied, with some researchers counting only the number of pecks and receiving pecks [21], while others have taken the interactions (i.e. agonistic and avoidance behaviour) between animals into account [19,20,22]. Nevertheless, there are no major differences between the animal rankings in groups when the different ranking methods are used. In most cases, the animals defined as 'pure' peckers or receivers in our study were also among the top ranking or bottom ranking animals using the previously proposed formulas to rank animals in groups [19-22]. These similarities indicate that the groupings used in this study represent aggressive and submissive animals.

### Genes and behaviour

The P&R group had great variability in terms of the number of pecks given and received and is large compared to both the R and the P groups (Figure 1). This may explain why there were no significant genes at the FDR *P *< 0.1 level for the P&R vs. R comparison (Additional File 1). The P&R vs. P comparison detected 58 significant genes, and of these 30 overlapped with significant genes from the R vs. P comparison. This suggests that in both of these cases, the gene expression differences were caused by the P group.

Interpreting the DE genes in the R vs. P comparison is a first step towards understanding the underlying genetic mechanism behind aggressive behaviour in chickens. The R vs. P comparison identified 40 significant genes. It was not fully clear how the annotated genes were involved in regulating aggressive behaviour; however, as the annotation of the chicken genome improves, this may provide a clearer picture of how the genes are related to aggressive behaviour. None of the most obvious candidate genes for aggressive behaviour [see [16]] were identified in our comparisons. For example, it was previously shown that serotonergic receptors play a role in aggression in chickens [15]. It is possible that these genes were up- or down-regulated in a specific part of the brain, but could not be detected in our study of whole brain gene expression because the effect was diluted. Another explanation is that these genes influence aggressive behaviour via different allelic forms.

The gene set enrichment analysis, which involved all of the genes on the array, demonstrated that genes involved with muscle development were among the significant GO identifiers. In *Drosophila *tested for aggressive behaviour, genes involved in muscle contraction were among the significant GO identifiers [23]. It is not clear whether genes related to muscle development have the same function in the brain; however, in chickens, the largest bird usually ranks the highest [24]. In spite of this, we observed no difference in body weight between the pecking and receiver animals in our study.

Other GO identifiers detected in this study were involved in lipid metabolism, lipid synthesis and low density lipo-protein binding (Figure 5). Fatty acid binding proteins have been shown to play a role in the differentiation of neurons and glial cells in rats [25]. As a consequence of improved neural development, subsequent changes in the brain and behaviour could occur between low and high ranking animals, such as differences in the development of memory functions. In our study, some of the significant GO identifiers coded for synaptosomes, glutamate receptor binding, and the regulation of excitatory postsynaptic membrane potentials. Glutamate receptors, which were detected in the GO identifiers, play an important role in the development of memory formation following passive avoidance training in young chickens [26]. Therefore, memory may play a role in remembering the social hierarchy of the group.

## Conclusion

In conclusion, our study provides a first insight into which genes are involved in aggressive behaviour in chickens. The results of our study showed that the level of expression is not dependent on the number of pecks given or received. It was not fully clear how the DE genes were involved in regulating aggressive behaviour. However, the gene set enrichment test showed that the DE genes coded for synaptosomes, and genes involved with lipid metabolism and memory formation. When confirmed in future studies, the DE genes may help scientists understand how the pecking order forms in laying hens at a molecular level.

## Methods

### Chicken lines, tissue sampling

In this study, we used 60 randomly selected laying hens from a high feather pecking selection line. This line was selected for eight generations for increased feather pecking behaviour based on a social feather pecking test, but the hens showed no difference in aggressive pecking behaviour when compared to the low feather pecking line [27]. The birds were reared in floor pens covered with a 5-cm thick layer of wood shavings. The temperature was 34°C when the hens were one day old, and it was gradually reduced to 20°C by the time the hens were eight weeks of age. The temperature was then maintained at 20°C for the remainder of the experiment. The light regime was 12 h light:12 h dark (12L:12D) from 0 to 14 weeks. Then one hour of light was added per week until the light regime was 16L:8D when the hens were 18 weeks of age. At 18 weeks, the chickens were transferred to four-bird battery cages in two levels. At 33 weeks of age, the chickens were randomly divided into three groups of 20 hens, and their body weights were measured. The birds had a week to adapt to the new environment and group composition. The groups consisted only of hens in order to replicate the commercial conditions that laying hens are maintained under. When the hens were 34 weeks of age, they were videotaped for 3 h between 14:00 h and 17:00 h. The 3 h time frame was chosen because this was the same time frame used during the selection procedure for the line. For each hen, the number of pecks given and received was scored. Aggressive pecks were defined as hard pecks aimed at the head or comb of the receiving bird, where no feather pulling was involved [5]. During the next day, between 8:00 h and 12:00 h, the 60 birds were decapitated. The whole brains were extracted and immediately frozen in liquid nitrogen and stored at -80°C for further use. The experiment has been performed according the regulation of the Danish Committee of Control with Animal Research (Dyreforsøgstilsynet).

### Expression profiling using microarrays

In total 60 samples from a high feather pecking line were used for the expression profiling experiments. The expression profiles of the 60 brains were measured using 20 K chicken oligo microarrays, which were printed and supplied by ARK-Genomics, Roslin Institute, UK via the European Animal Disease Genomics Network of Excellence (EADGENE) consortium. The arrays contained 20,678 oligos (64 to 70 mers), which corresponded to 20,640 chicken transcripts based on UMIST full length cDNA, DT40 full length cDNA, and ENSEMBL and TIGR ESTs Contigs http://bioinformatics.roslin.ac.uk/eadgene/index.php/Chicken_-_Genomic_resources. More detailed descriptions of the 20 K chicken oligonucleotide microarrays are available at the National Center for Biotechnology Information's (NCBI's) Gene Expression Omnibus (GEO) [28,29], which is available through the GEO platform accession number GPL5480. Dual-channel microarray experiments were performed with a common reference design using total brain RNA that was purified from an unrelated animal as the reference. During the experiment, care was taken not to confound the factors of interest with the experimental batch sets. The whole brain was homogenized in liquid nitrogen using a TissueLyser (Qiagen-Retsch GmbH, 42781, Haan, Germany) fitted with 50 mL stainless steel grinding jars and 20 mm grinding balls. The total RNA was purified and treated with DNase treated using NucleoSpin RNA L (Macherey-Nagel GmbH & Co KG, 52355, Düren, Germany) following the enclosed protocol. The purified RNA samples were quantified using a NanoDrop ND-1000 spectrophotometer (NanoDrop Technologies, Thermo Fisher Scientific, Wilmington, DE 19810, USA), and the quality was evaluated by agarose gel electrophoresis. The samples were then stored at -80°C until use. From each sample, 10 μg of total RNA was labelled with Alexa-647, and 10 μg of the reference sample was labelled with Alexa-555 using the SuperScript Direct cDNA labelling System (Invitrogen, 2630, Taastrup, Denmark). The labelled cDNA was purified using the NucleoSpin 96 Extract II PCR Clean-up kit (Macherey-Nagel GmbH & Co KG, 52355, Düren, Germany). The labelled reference samples were mixed and divided into aliquots before combining the reference aliquots with the labelled samples. The slides were hybridised in six batches using a Discovery XT hybridization station (Ventana Discovery Systems, Tucson, AZ, USA) followed by scanning at a 5 μm resolution using the ScanArray Express HT system (version 3.0, Perkin Elmer, Waltham, MA, 02451, USA). Image analysis was conducted using GenePix Pro (version 6.0.1.27, Molecular Devices, Sunnyvale, CA, 94089-1136, USA) using irregular filled feature types and "MorphologicalClosingFollowedByOpening" background values. More detailed descriptions of the microarray experiment and data are available at the NCBI's GEO [28,29] through the accession number GSE10380.

Statistical analysis of the microarray data was carried out in the R computing environment (version 2.5.0 for Windows) using the Linear Models for Microarray Analysis package (Limma, version 2.10.0, [30]), which is part of the Bioconductor project [31]. Spots flagged as "Not found" ("Flags" = -50) by GenePix Pro and spots with either a "SNR 647" or "SNR 555" value less than 1 were excluded from the analysis by assigning the spot a weight of zero. The log_2_-transformed ratios of Alexa-647 to Alexa-555 (not background corrected median values) underwent within-slide normalizations using weighted loess with default parameters.

### Statistical analysis

The statistical analysis was performed in six steps. In Step 1, we tested for differences between cages in the level of aggressive pecking performed and received using the Kruskal-Wallis rank sum test (kruskal.test option in the R version 2.5.0). Differences in body weight between the cages and the rank order groups (see Step 2) were calculated using an analysis of variance (ANOVA) test.

In Step 2, we first examined the Spearman correlation between the expression level of each gene (M value) and the number of aggressive pecks given or received to test for equality to zero. The test was performed for 15,242 genes, and less than 30 values were missing. The *P *values were adjusted for multiple testing using the FDR procedure [32]. Second, the animals were grouped based on a combination of the number of aggressive pecks performed and received. The grouping of the animals may be performed in many different ways. From a practical point of view, we were interested in what made some birds peck (aggressive) and what made some birds be pecked (victim/submissive). Therefore, the birds were assigned into groups based on a combination of the 'number of pecks performed' and the 'number of pecks received'. We assumed that the number of pecks performed was partly regulated by genes and that being a victim was regulated by different genes. This allowed the animals to be categorized into a group of 'pure' peckers and a group of 'pure' receivers (victims), which may show the difference between the peckers (P) and receivers (R). The group of birds which performed both pecking and receiving (P&R) was considered an intermediate. Group 1 contained animals that both pecked and received pecks. Group 2 contained animals that received pecks, but did not peck themselves. Group 3 contains animals that pecked, but did not receive any pecks. Lastly, Group 4 contained animals that did not peck and did not receive any pecks. Comparisons were made between Groups 1 and 2 (P&R vs. R), between Groups 1 and 3 (P&R vs. P), and between Groups 2 and 3 (R vs. P) using t-tests.

In Step 3, the differential expression of each gene was assessed using linear modelling and empirical Bayes methods, which were implemented using the R package Limma [30]. Test-pen was a fixed factor in the model. Each transcript targeted by a probe was tested for its expression change using a modified t-test. In the modified t-test, the residual standard deviations are moderated across the probe sets to ensure that there is a more stable inference for each transcript. The moderated standard deviations are a compromise between the individual transcript-wise standard deviations and the pooled standard deviation. Genes with an adjusted *P *value (FDR) < 0.1 are reviewed in the discussion, while the genes with *P *values < 0.01 are presented in Additional Files 1, 2 &3.

In Step 4, the significant genes (FDR *P *< 0.10) were studied using a 2D-cluster analysis using the heatmap2 function from the gplots library (version 2.3.2) http://cran.r-project.org/web/packages/gplots/gplots.pdf. The genes were clustered based on their normalized expression values using the correlation method.

In Step 5, the features on the arrays were annotated. For the annotations, we used 1) an annotation file available at http://www.sigenae.org/fileadmin/_temp_/EADGENE_annotation/V2/EADGENE_oligo_annotation_GO_chicken_V2.csv, and 2) a Unigene identifier, which was used to map the features on the array, and an annotation package, which was built using the Bioconductor package AnnBuilder (version 1.14.0).

In Step 6, each gene on the array was assigned to a GO identifier, and a gene set enrichment test was performed to compare P&R vs. P and R vs. P using Limma [30,33]. This is a modified version of the gene set enrichment test reported by Mootha et al [34]. For this test, it is not necessary to make a hard cut-off point between the genes that are DE and those that are not [34]. The method is especially useful in for traits that are influenced by many genes, which each have a small effect, like behavioural traits.

## Authors' contributions

Conceived and designed the experiment: BB. Performed the microarray experiment: JH. Analyzed the data: BB, PS, and LJ. Wrote the paper: BB. All authors contributed to the discussion of the results and agreed on the contents of the paper.

## Supplementary Material

Additional file 1 **Table S1**. Differentially expressed genes between the pecker and receiver (P&R) group and the receiver (R) group Click here for file

Additional file 2 **Table S2**. Differentially expressed genes between the pecker and receiver (P&R) group and the pecker (P) group Click here for file

Additional file 3 **Table S3**. Differentially expressed genes between the receiver (R) and the pecker (P) group Click here for file

Additional file 4 **Heatmap of differentially expressed genes between the pecker and receiver (P&R) group and the pecker (P) group**. There were 58 differentially expressed genes (FDR *P *< 0.01). The M values of the genes (rows) were ordered using the centred Pearson correlation and hierarchical clustering. The dendrogram shows the clustering results of the gene expression profiles. The arrays (columns) represent the individual hens, which are denoted with their id number, number of aggressive pecks performed, and number of aggressive pecks received (id_# pecks performed_# pecks received). The dendrogram shows the clustering results of hens based on the gene expression profiles. The red and green colours denote high and low intensities, respectively. Click here for file

Additional file 5 **Representation of the significant GO identifiers detected in the comparison between the pecker & receiver (P&R) group and the pecker (P) group**. The three alternatives (mixed, up, and down) are represented in the columns. The "mixed" alternative tested whether the genes in the set tended to be differentially expressed, without regard for the direction. In this case, the test is significant if the set mainly contains large test statistics, even if some results are positive and some are negative. The "up" alternative tested whether the genes in the set were up-regulated. The "down" alternative tested whether the genes in the set were down-regulated. The description of the biological pathways are listed in the rows and the GO class is listed in brackets (BP: biological processes, CC: cellular components, MF: molecular function). The colour gray denotes the GO identifiers *P *> 0.01. Click here for file
