# Supplementary figures and images for: Differentially expressed genes for aggressive pecking behaviour in laying hens
Source: BMC Genomics. 2009 Nov 19;10:544. doi: 10.1186/1471-2164-10-544 (PMC2785841; doi:10.1186/1471-2164-10-544)

Color Key

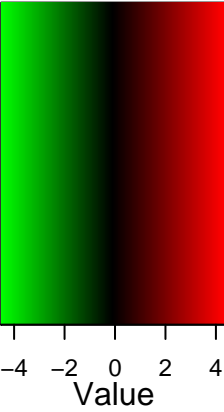

P&R versus P

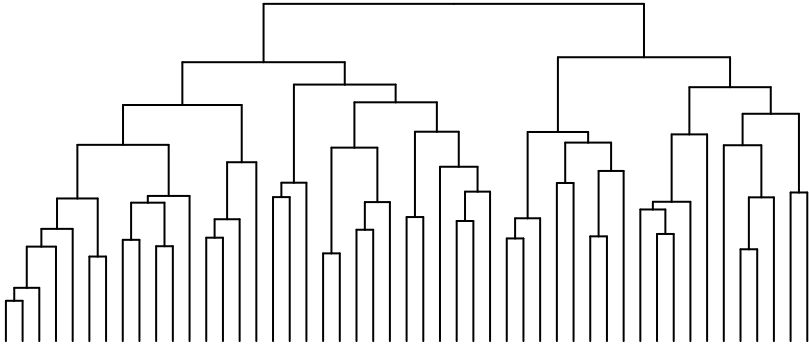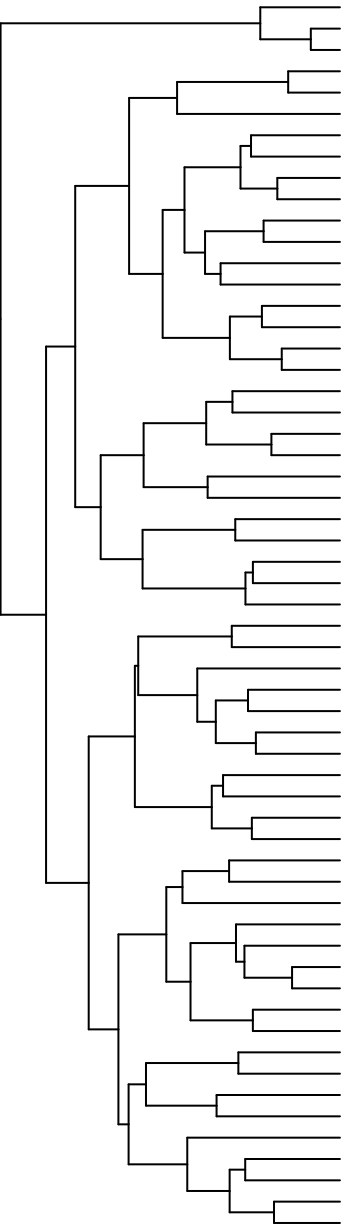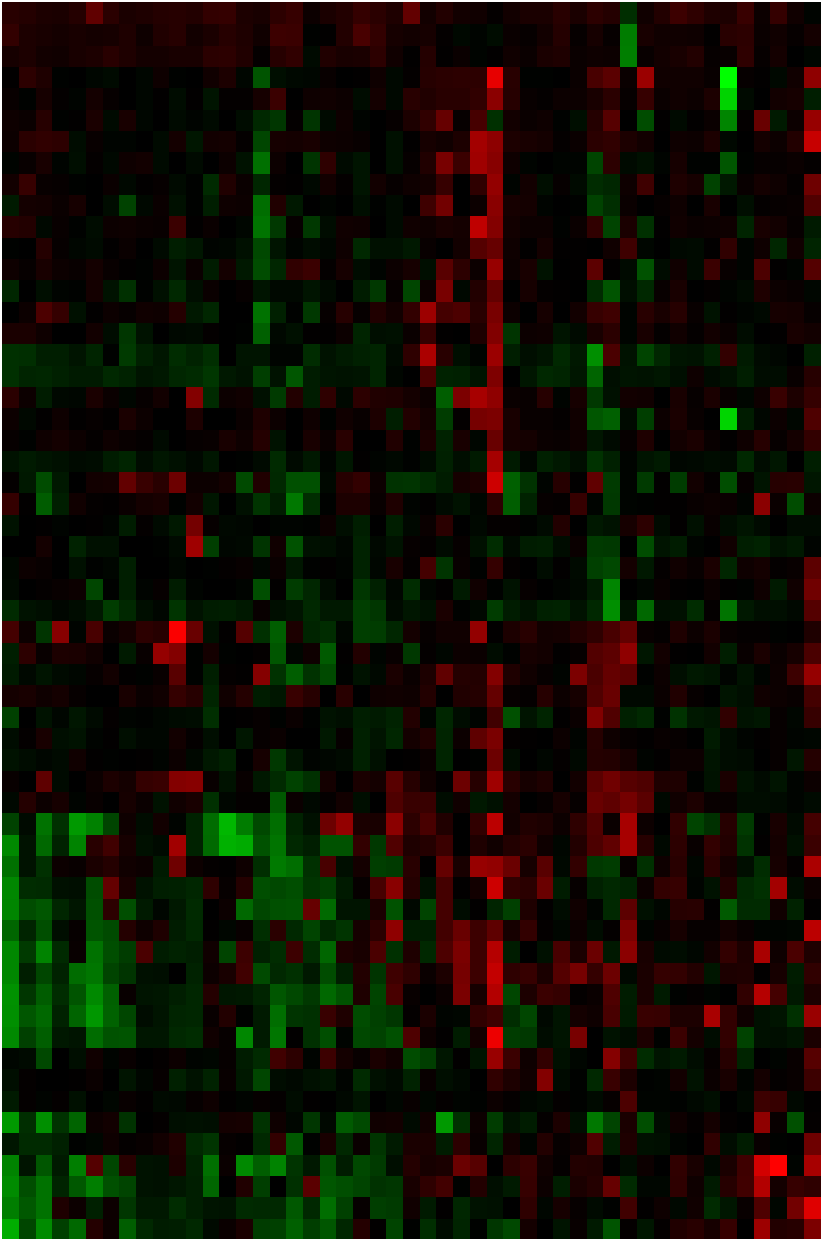

4097\_5\_6  
4042\_13\_6  
4082\_15\_4  
4204\_1\_8  
4173\_8\_6  
4009\_5\_4  
4065\_2\_46  
4165\_1\_2  
4233\_1\_7  
4093\_2\_4  
4133\_6\_5  
4228\_14\_0  
4248\_1\_1  
4269\_14\_1  
4053\_4\_2  
4285\_1\_5  
4286\_2\_8  
4252\_6\_4  
4294\_1\_3  
4050\_1\_8  
4109\_3\_3  
4124\_3\_12  
4147\_18\_2  
4015\_3\_2  
4153\_8\_5  
4074\_1\_3  
4292\_1\_2  
4045\_3\_4  
4205\_7\_6  
4056\_7\_0  
4126\_3\_4  
4028\_4\_5  
4246\_1\_6  
4130\_1\_4  
4283\_1\_7  
4218\_3\_9  
4262\_2\_4  
4156\_1\_7  
4011\_3\_7  
4105\_4\_3  
4112\_5\_6  
4307\_1\_10  
4100\_1\_2  
4116\_11\_5  
4250\_3\_4  
4240\_22\_0  
4231\_9\_6  
4061\_9\_5  
4036\_13\_0

Supplement: Additional file 4 — Heatmap of differentially expressed genes between the pecker and receiver (P&R) group and the pecker (P) group. There were 58 differentially expressed genes (FDR P < 0.01). The M values of the genes (rows) were ordered using the centred Pearson correlation and hierarchical clustering. The dendrogram shows the clustering results of the gene expression profiles. The arrays (columns) represent the individual hens, which are denoted with their id number, number of aggressive pecks performed, and number of aggressive pecks received (id_# pecks performed_# pecks received). The dendrogram shows the clustering results of hens based on the gene expression profiles. The red and green colours denote high and low intensities, respectively. [file 1471-2164-10-544-S4.PDF]

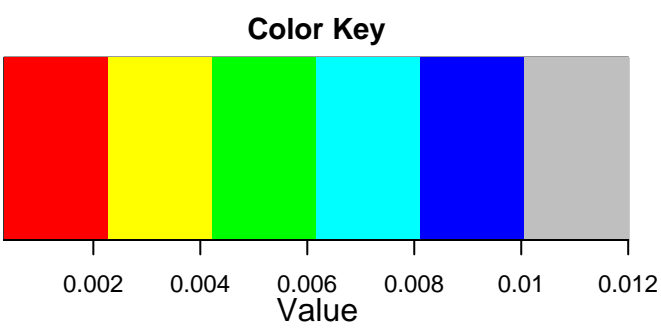

P&R-P

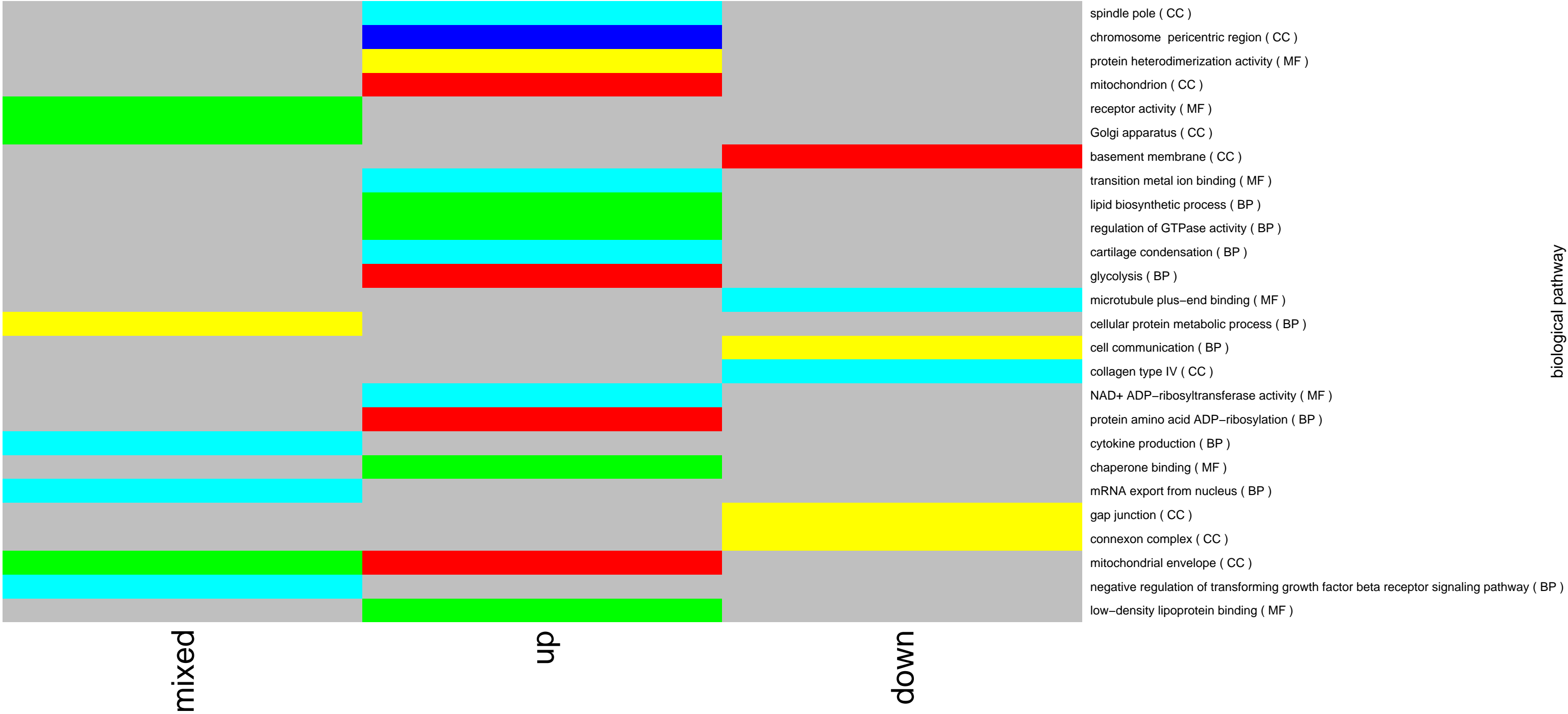

Supplement: Additional file 5 — Representation of the significant GO identifiers detected in the comparison between the pecker & receiver (P&R) group and the pecker (P) group. The three alternatives (mixed, up, and down) are represented in the columns. The "mixed" alternative tested whether the genes in the set tended to be differentially expressed, without regard for the direction. In this case, the test is significant if the set mainly contains large test statistics, even if some results are positive and some are negative. The "up" alternative tested whether the genes in the set were up-regulated. The "down" alternative tested whether the genes in the set were down-regulated. The description of the biological pathways are listed in the rows and the GO class is listed in brackets (BP: biological processes, CC: cellular components, MF: molecular function). The colour gray denotes the GO identifiers P > 0.01. [file 1471-2164-10-544-S5.PDF]
